# Ambient particulate air pollution (PM2.5) is associated with the ratio of type 2 diabetes to obesity

**DOI:** 10.1038/s41598-017-08287-1

**Published:** 2017-08-22

**Authors:** Mohsen Mazidi, John R. Speakman

**Affiliations:** 1 State Key Laboratory of Molecular Developmental Biology, Institute of Genetics and Developmental Biology, Chinese Academy of Sciences, Chaoyang Beijing, China; 2 University of the Chinese Academy of Sciences, Beijing, China; 3 Institute of Biological and Environmental Science, University of Aberdeen, Scotland, UK

## Abstract

We used county level data for T2D prevalence across the mainland USA and matched this to county level ambient PM2.5. Multiple linear regression was used to determine the relation between prevalence of T2D with PM2.5 after adjustment for confounding factors. PM2.5 explained 6.3% of the spatial variation in obesity, and 17.9% of the spatial variation in T2D. After correcting the T2D prevalence for obesity, race, poverty, education and temperature, PM2.5 still explained 8.3% of the residual variation in males (P < 0.0001) and 11.5% in females (P < 0.0001). The effect on obesity prevalence corrected for poverty, race education and temperature was much lower and hence the ratio of T2D to obesity prevalence was significantly associated with PM2.5 in males (R^2^ = 11.1%, P < 0.0001) and females (R^2^ = 16.8%, P < 0.0001). This association was repeated across non-African countries (R^2^ = 14.9%, P < 0.0001). High levels of PM2.5 probably contribute to increased T2D prevalence in the USA, but have a more minor effect on the obesity. Exposure to high environmental levels of PM2.5 (relative to the USA) may explain the disproportional risk of T2D in relation to obesity in Asian populations.

## Introduction

Worldwide obesity has nearly doubled since 1980 and high body mass index (BMI) is now recognized as one of the most important determinants of global disease burden^1, 2^. In the same vein, the prevalence of type 2 diabetes (T2D) is increasing rapidly all over the world, with a predicted 592 million cases by the year 2035^3^. The combination of continued increase in prevalence of diagnosed diabetes with declining overall mortality have led to an acceleration of lifetime risk, and more years spent with diabetes^4^. In North America, the 2010 Global Burden of Disease Study ranked high BMI as the second most important risk factor for disease burden behind tobacco smoking^1^. A report from the American Diabetes Association confirmed that diabetes is a major factor in total economic costs in the U.S, totalling $245 billion in 2012 and accounting for 1 in every 10 health care dollars^5^. The T2D epidemic has been attributed to enhanced accessibility of unhealthy foods, sedentary lifestyles and environmental risk factors, such as air and land pollution^6^, which can lead to the occurrence of overweight or obesity. Although obesity and T2D are clearly related to one another the ratio of the prevalence of T2D to obesity varies widely between different countries. In the USA for example, there are approximately 3 people with obesity for every person with T2D. In China and India, however, these numbers are reversed, with more than 2 people with T2D for each person with obesity. The causes of these different ratios remain obscure.

Airborne particulate matter (particles with diameters of 2.5 μm or less, known as a PM2.5) has consistently been shown to increase mortality and morbidity^7, 8^. In this regard, the World Health Organization (WHO) stated that air pollution is the world’s largest single environmental health risk^9^. As a main component of haze, smoke, and motor vehicle exhaust, PM2.5 is dangerous in part because of its small size and ability to invade critical human organs in the respiratory and vascular systems^10^. Although the major diseases associated with particulate matter include respiratory disorders such as asthma, chronic obstructive pulmonary disease (COPD) and lung cancer^11, 12^ as well as cardiovascular problems^13^ there is growing evidence suggesting that small particulates are an important risk factor for T2D^14–17^. Several previous studies have shown a positive association of air pollution and T2D risk^18–20^, however other studies indicated no significant effect^21, 22^ or with borderline significance^23^. Several recent meta-analyses reported a high risk of bias for the available studies^24^ and they suggested that future studies should take more account of confounding factors such as socioeconomic variables^24^ like poverty and race, Although a single previous study investigated the spatial distribution of PM2.5 levels across the mainland USA, and their association with the spatial distribution of T2D^25^ this study did not take into account the important potential confounding impacts of temperature.

In a recent paper, we have highlighted the spatial association between T2D prevalence and ambient temperature, across the mainland USA^26^. The prevalence of T2D in the USA is higher in areas that are warmer, even when confounding factors such as obesity levels, poverty and race are taken into account. This could be associated with the stimulatory effects of cold on brown adipose tissue^27^ which is a major disposal site for ingested glucose^28^. There is considerable evidence suggesting that PM2.5 is affected by meteorological factors such as ambient temperature, relative humidity and wind direction/speed^29, 30^, although the relationship is complex, and probably dependent on the temporal and spatial scale at which the association is considered. This complexity arises because different components of the PM2.5 react to ambient temperature changes in different ways^31^. Hence, sulphate particles in PM2.5 generally increase as temperature increases, because of increased oxidation of Sulphur Dioxide (SO_2_), while nitrate particles in PM2.5 are independent of, or negatively associated with temperature, because nitrates shift from the particle phase to the gaseous phase as temperature rises^32, 33^. On balance, however, across the entire mainland USA, at the spatial scale of 2.5° × 2.5° blocks, total PM2.5 is positively associated with ambient temperature^30^, presumably because PM2.5 across the USA is dominated by sulphate particles. This is also true for temporal variation over months and years in the Mediterranean^29^, but in India (Dehli) the opposite is the case^34^. Since in our previous study we did not account for PM2.5 levels as a potential confounder, and also as the previous study of spatial distribution of PM2.5 effects did not account for temperature, this raises three potential scenarios. First, our inference of an association of temperature and T2D prevalence may have been an artefact of not accounting for the PM2.5 effect. Second, the previous inference of an association of PM2.5 levels and T2D may have been an artefact of not accounting for the confounding effects of temperature (and poverty). Alternatively, both ambient temperature and PM2.5 may have independent associations with T2D prevalence. In the current paper we aimed to resolve which of these three scenarios is most likely, using the publicly available county level data across the mainland USA. In addition if PM2.5 affects T2D prevalence, but not obesity, then one would anticipate that the ratio of T2D to obesity should rise as the levels of PM2.5 increase. This might then explain the much higher ratio of T2D to obesity in China and India, than in the USA. Hence we sought to use the same county level data in the USA to explore the association between PM2.5 and the ratio of T2D to obesity, and then expanded this analysis to explore the global association between PM2.5 and the same ratio using country level data from across the world.

## Methods

Preparation, definition, download and sorting of data on prevalence of obesity, T2D, poverty, race and temperature have been explained elsewhere^26, 35^. Briefly, we downloaded the county level data on the age adjusted prevalence and incidence of obesity and T2D from the USA Centers for Disease Control and prevention web site (www.cdc.gov). The prevalence of diabetes and obesity (2010) was estimated using data from the CDC Behavioural Risk factor surveillance system (BRFSS) which is a monthly state based telephone survey of a nationally representative sample of adults aged >20 years old. Because it is telephone based it excludes individuals living in care homes or those without a telephone. More than 400,000 individuals are contacted annually to take part in the survey which has been running since 1984. Individuals are judged to have diabetes if they respond ‘yes;’ to the question “Has a doctor ever told you that you have diabetes?”, excluding females who indicate in a follow-up question that they only had diabetes during pregnancy. Previous work indicates that self-report of a physician’s prior diagnosis of diabetes is highly reliable compared to medical records^36^. This question does not separate those with type 1 and type 2 diabetes. In the adult population of the USA more than 96% of diabetes is type 2, we therefore called the estimated prevalence that of T2D. Given the magnitudes of the trends described in the present paper, they cannot be attributed to differences in prevalence of the type 1 diabetes. For obesity, in the telephone interview, individuals self-report their height and weight in response to the questions “About how much do you weigh without shoes?” and “About how tall are you without shoes?” which are then converted if necessary to kg and metres before calculating the Body mass index (BMI = (height)^2^/weight). A BMI > 30 is then classed as obese using the WHO standard for Caucasians^37^. This is applied independent of actual race. Individuals normally over estimate their own height and underestimate their own weight in a self-report setting^38, 39^ and hence these estimates are likely to be conservative. However, even though individuals may underreport their body weight, this is unlikely to be biased with respect to their exposure to PM2.5 levels. In addition to the health questions individuals are also asked a core of demographic questions which include age, sex, race, marital status, education, employment status, and income and home ownership status. The data were available for 3106 counties or county equivalents from the continental USA, reflecting a population of around 170 million adults. A previous variogram analysis (26) showed that the county level data is a suitable spatial scale at which to seek disease associations for T2D and obesity. The correlation of incidence to prevalence was 0.98 for T2D. Here we report only the relationships for prevalence, as the relationships for incidence were very similar. Oak Ridge National laboratory was the source of the ambient temperature data (http://www.daac.ornl.gov: files B01, B02 and C07), and the records of county level poverty (% in poverty and average income) and racial make-up data from the United States Census Bureau, 2010 census data (http://www.census.gov/2010census specifically files PVY01, PVY02, INC01, INCO2, INCO3, IPE01, RHI02). Estimates of PM2.5 for each county were obtained for 2003–2011 as documented on CDC WONDER (http://wonder.cdc.gov), specifically file D80a. Reported measures are the average daily estimates of PM2.5 over the 7 year interval in micrograms per cubic meter (µg.m^−3^). The values are summarized to the county-level, from 10 kilometer square spatial area grids covering the 48 contiguous United States (not including Alaska and Hawaii). Two sources of environmental data were used as input to the surfacing algorithm, US Environmental Protection Agency (EPA) Air Quality System (AQS) PM2.5 *in-situ* data and National Aeronautics and Space Administration (NASA) Moderate Resolution Imaging Spectroradiometer (MODIS) aerosol optical depth remotely sensed data. We combined the estimated prevalence data for obesity and T2D with the PM2.5 records for each county (identified by the Federal information processing standard (FIPS) code), and the records of county level poverty (% in poverty), racial make-up (% African American), education (% with bachelor’s degree or higher), and temperature. Since data were not available for all parameters for all counties this left a slightly smaller total sample for some analyses. These data allowed us to normalise the prevalence data at the county level for these confounds and then examine the associations of PM2.5 to the obesity prevalence corrected for race, poverty, education and temperature. In case of the obesity, multiple linear regression was used to determine its relation between with PM2.5, corrected for race, poverty, education and temperature. The association was determined for each sex independently. For T2D, we also used multiple linear regression to estimate the association between T2D and PM2.5 corrected for obesity, race, poverty, education and temperature.

For prevalence of the T2D globally, we have obtained the data from “The World Bank” (http://www.worldbank.org/) database. In this database, prevalence refers to the percentage of people ages 20–79 that have type 1 or type 2 diabetes and the source is International Diabetes Federation. Also for the national PM2.5 levels “The World Bank” database was used, in this database.

PM2.5 pollution is defined as the average level of exposure of a nation’s population to concentrations of suspended particles measuring less than 2.5 microns in aerodynamic diameter. Mean annual concentrations of PM2.5 by population in both urban and rural areas is reported^40^. For the prevalence of the obesity, the report “Global Burden of Disease Study” was used. This defined obesity as a BMI ≥ 30 kg/m^2^ ^41^. For analysis we removed the data for small island states with populations less than 1 million individuals. In addition, we also removed one outlier (Vietnam) with an exceptionally high diabetes to obesity ratio (4.0) calculated from very low values of disease prevalence for both diseases. SPSS software (version 11.5, Chicago, IL, USA) was used for statistical analysis. A P-value ⩽0.05 was considered statistically significant.

## Results

### Obesity

**Figure 1:**
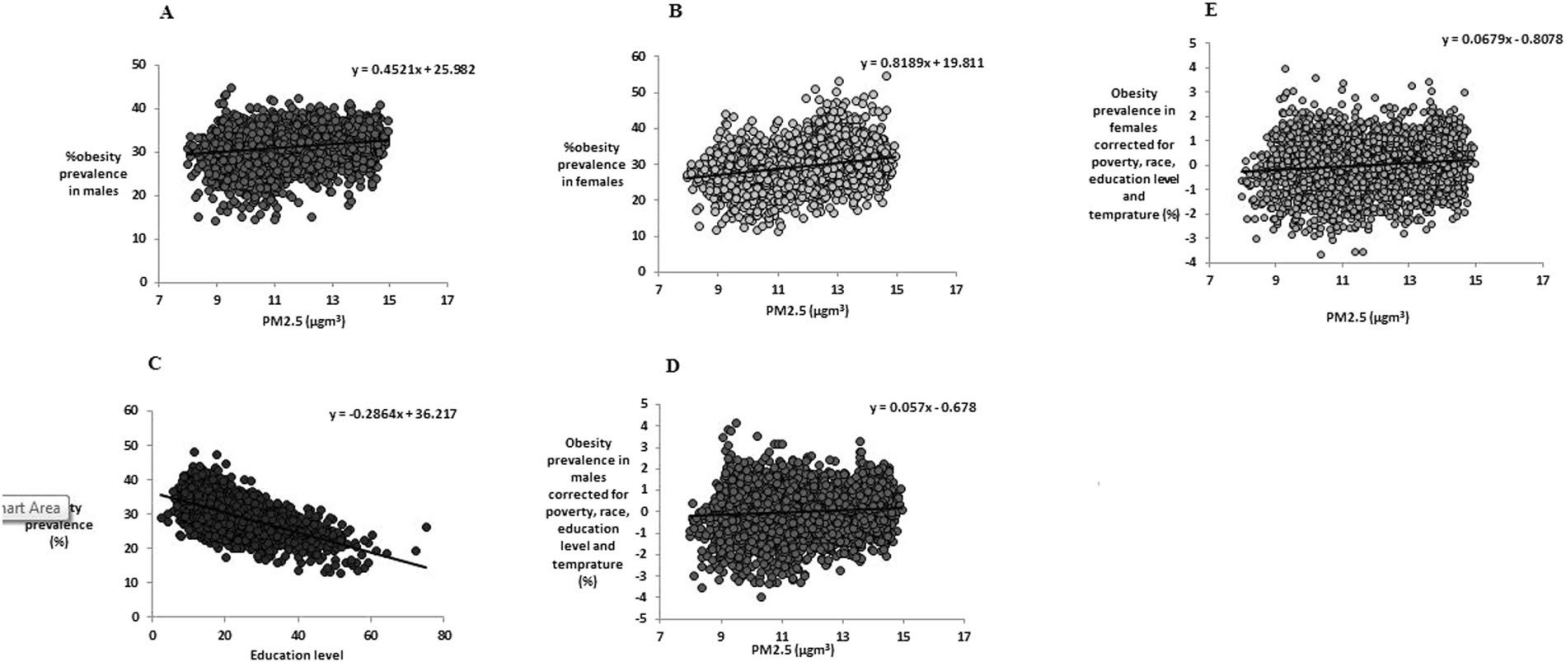
Association between levels of obesity prevalence for each sex and PM2.5 across the mainland USA. Plots show the county level data (n = 3106 counties) for (**A**) obesity prevalence and average PM2.5 (µg.m^−3^) for males, (**B**) obesity prevalence and average PM2.5 (µg.m^−3^) for females, (**C**) association between obesity prevalence and education level, (**D**) the association between obesity prevalence corrected for race, poverty, education and temperature and average PM2.5 (µg.m^−3^) for males, (**E**) the association between obesity prevalence corrected for race, poverty, education and temperature and average PM2.5 (µg.m^−3^) for females.

There was a positive relationship between obesity prevalence and PM2.5 for each sex (males: R^2^ = 0.041, F_1, 3106_ = 131.9; females: R^2^ = 0.076, F_1, 3106_ = 255.7, P < 0.0001 for both, Fig. 1A,B). With respect to confounding factors for obesity, we reported previously^26^ that there were significant positive associations between obesity prevalence and both poverty (%) and the percent of the population in a county that was African American (%) (R^2^ = 0.254 and R^2^ = 0.159 respectively, P < 0.0001 for both)^26^. In addition, we found here that there was a strong negative relationship between obesity prevalence and education (R^2^ = 0.349, P < 0.0001 Fig. 1C). When we corrected the obesity prevalence for race, poverty, education and temperature there was still a significant relationship with PM2.5 but the explained variance was much lower in both males (R^2^ = 0.01, F_1,3104_ = 41.0, P < 0.0001) and females (R^2^ = 0.014, F_1,3104_ = 42.6, P < 0.0001) (Fig. 1D,E). When we corrected the obesity prevalence for race, poverty, education and PM2.5, ambient temperature did not have impact on obesity prevalence in females (R^2^ = 0.0001, F_1,3104_ = 0.18, P = 0.669) but there was a significant effect in males. Nevertheless the explained variance in obesity rates by temperature in males was extremely low (R^2^ = 0.006, F_1, 3104_ = 19.7, P < 0.0001). These data suggest that PM2.5 does have a weak association with obesity even when temperature and socioeconomic factors are taken into account, but in accord with our previous study, which considered pooled data across the sexes (26), the association of ambient temperature to obesity is extremely weak or absent.

### Diabetes

**Figure 2:**
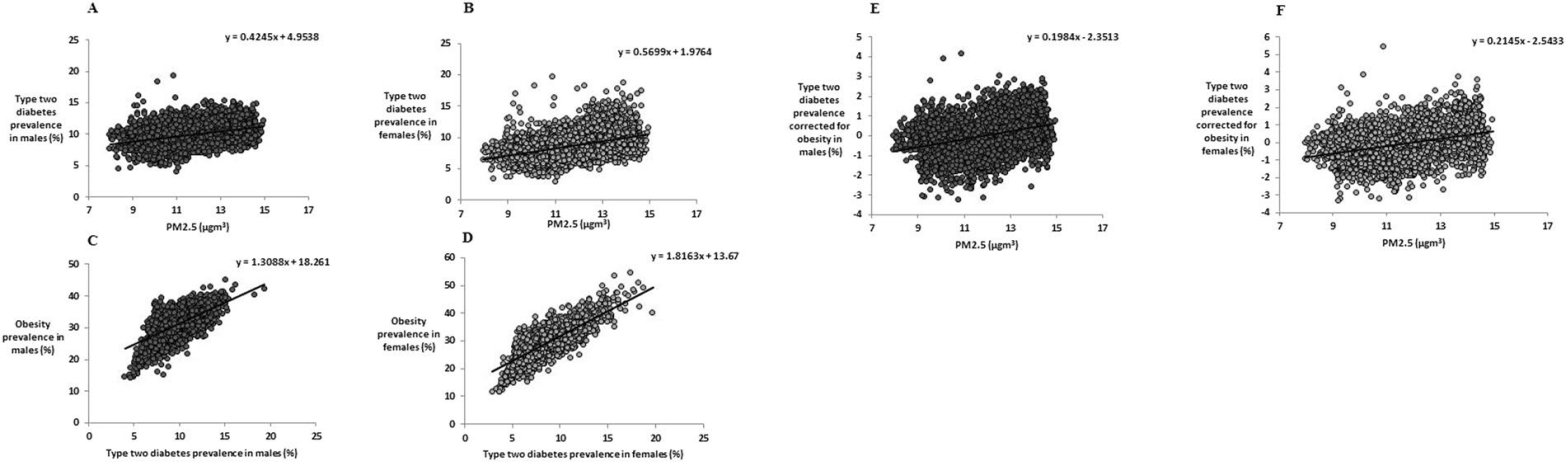
Association between levels of T2D prevalence for each sex and PM2.5 across the mainland USA. (**A**) T2D prevalence and average PM2.5 (µg.m^−3^) for males, (**B**) T2D prevalence and average PM2.5 (µg.m^−3^) for females, (**C**) association between T2D prevalence and obesity prevalence for males and (**D**) association between T2D prevalence and obesity prevalence for females and (**E**) the association between T2D prevalence corrected for obesity and average PM2.5 (µg.m^−3^) for males, (**F**) the association between T2D prevalence corrected for obesity and average PM2.5 (µg.m^−3^) for females.

Using the age adjusted data there was a significant positive relationship between the prevalence of T2D and PM2.5 for each sex (males: R^2^ = 0.154, F_1,3105_ = 566.9; females: R^2^ = 0.197, F_1,3105_ = 760.0, P < 0.0001 for both, Fig. 2A,B). As anticipated there was a strong association between the prevalence of T2D and the prevalence obesity for both males (R^2^ = 0.401) and females (R^2^ = 0.613) (Fig. 2C,D). After we corrected the prevalence of T2D for the prevalence of obesity, for each sex, the PM2.5 still explained 11.6% (F_1,3105_ = 408.4, P < 0.0001) and 13.7% (F_1,3105_ = 493.4, P < 0.0001) of the residual variation in T2D prevalence in males and females, respectively (Fig. 2E,F). With respect to confounding factors for type 2 diabetes, we previously have shown^26^ that there were significant positive associations between both poverty (%), and the percentage of a given county that was African American (%) and type 2 diabetes prevalence (R^2^ = 0.373 and R^2^ = 0.383 respectively, P < 0.0001 for both)^26^. Here we add that there was a strong negative relationship between T2D prevalence and education (R^2^ = 0.182, P < 0.0001).

**Figure 3:**
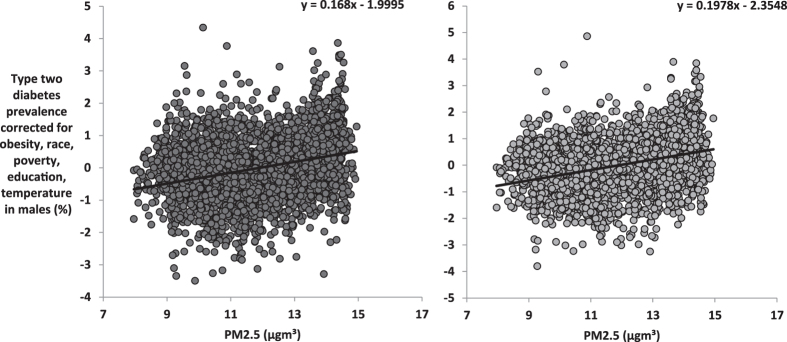
Association between levels of PM2.5 and T2D prevalence across the mainland USA corrected for obesity, race, poverty, education and temperature. Plots show the county level data across the USA (n = 3106) for (**A**) T2D prevalence corrected for levels of obesity, race, poverty, education and temperature against average PM2.5 (µg.m^−3^) in males, (**B**) T2D prevalence corrected for levels of obesity, race, poverty, education and temperature against average PM2.5 (µg.m^−3^) in females.

**Figure 4:**
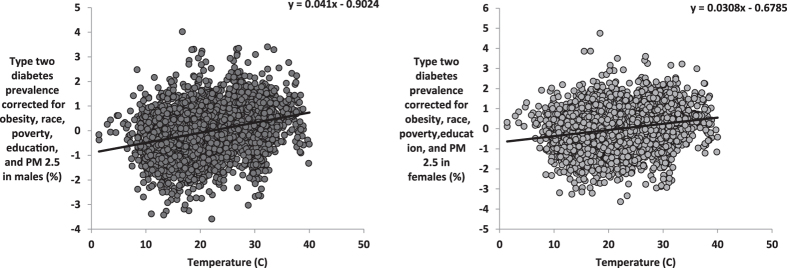
Association between temperature and T2D prevalence across the mainland USA corrected for levels of obesity, race, poverty, education and PM2.5. Plots show the county level data across the USA (n = 3106) for (**A**) T2D prevalence corrected for levels of obesity, race, poverty, education and PM2.5 (µg.m^−3^) against average temperature in males, (**B**) T2D prevalence corrected for levels of obesity, race, poverty, education and PM2.5 (µg.m^−3^) against average temperature in females.

When we corrected the T2D prevalence for obesity, race, poverty, education and temperature, PM2.5 still explained 8.3% of the residual variation in T2D prevalence in males (F_1,3105_ = 281.3, P < 0.0001, Fig. 3A) and 11.5% in females (F_1,3104_ = 404.4, P < 0.0001, Fig. 3B). When the T2D prevalence rates were corrected for obesity, race, poverty, education and PM2.5 and ambient temperature was used as the predictor, temperature explained 8.3% (F_1,3104_ = 281.6, P < 0.0001) and 4.7% (F_1,3104_ = 153.1, P < 0.0001) of the residual variation in T2D prevalence for males and females, respectively (Fig. 4A,B). Hence, both ambient temperature and PM2.5 had independent effects on T2D. To test if these effects were additive we included the interaction between these two factors into the model. There was a weak but significant interaction effect on T2D prevalence (β = −0.311, P = 0.038). The effect of PM2.5 was exaggerated at lower ambient temperature. There was also a significant interaction between the obesity prevalence and PM2.5 levels in their effects on T2D prevalence (β = 0.903, P < 0.0001).

**Figure 5:**
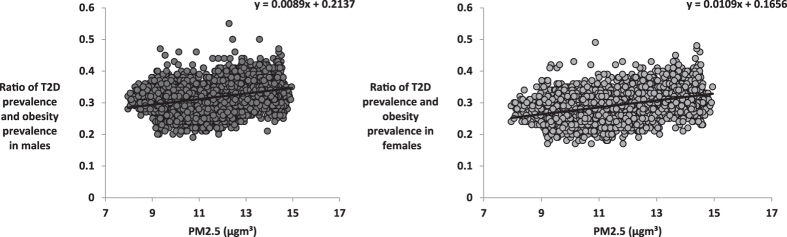
Association between levels of PM2.5 and ratio of T2D prevalence and obesity prevalence. Plots show the county level data across the USA (n = 3106), and global for (**A**) ratio of T2D prevalence and obesity prevalence against average PM2.5 (µg.m^−3^) for males, (**B**) ratio of T2D prevalence and obesity prevalence (T2D/Ob) against average PM2.5 (µg.m^−3^) for females.

**Figure 6:**
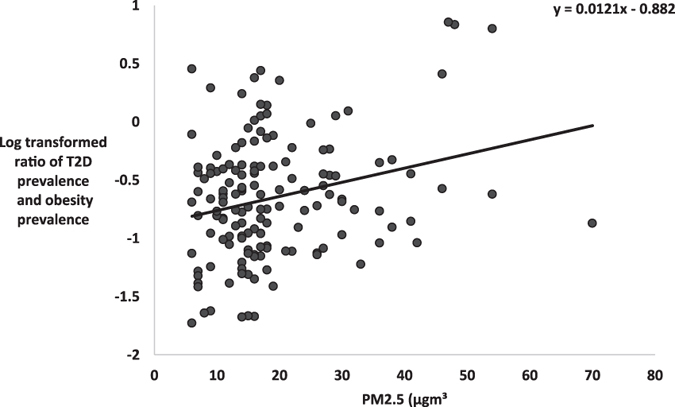
Ratio of T2D prevalence to obesity prevalence (T2D/Ob) against average PM2.5 (µg.m^−3^) across 146 countries.

The ratio of T2D prevalence to obesity prevalence (T2D/Ob) was significantly associated with PM2.5 in males (R^2^ = 0.111, F_1, 3105_ = 388.8, P < 0.0001, Fig. 5A) and females (R^2^ = 0.168, F_1, 3105_ = 627.0, P < 0.0001, Fig. 5B). We expanded this analysis to establish the association between T2D/Ob and PM2.5 levels at the country level across the world. There was a significant association between PM2.5 and the logged ratio of type 2 diabetes to obesity explaining 6.6% of the global spatial variation of the T2D/Ob ratio (F_1, 145_ = 2.07, P = .002, Fig. 6).

## Discussion

The results of the present study indicated that exposure to particulate air pollution (PM2.5) was significantly associated with prevalence of T2D, even after adjustment for potential confounding factors including obesity levels, socioeconomic factors such as race, poverty and education, and other environmental factors such as ambient temperature. In addition, ambient temperature also remained a significant factor influencing T2D prevalence when obesity, socioeconomic factors and PM2.5 were taken into account, reinforcing our previous finding (26) and suggesting PM2.5 and ambient temperature have independent effects on T2D prevalence. These patterns were repeated in both sexes (and were also found for T2D incidence: analysis not shown).

Previous work has been inconclusive with respect to the impacts of PM2.5 on T2D risk. A study that explored the prevalence and incidence of T2D with long-term exposure to PM2.5 at 6 US sites (5,839 participants, aged 45–84 years) reported that PM2.5 was not associated with incident T2D over the course of the 9-year follow-up^42^. Moreover, in a study focussed on African American women living in 56 metropolitan areas across the US, there was little support for an association of PM2.5 with T2D incidence^43^. Our analyses are not consistent with these previous studies. However, the current data are consistent with previous studies that suggested exposure to air pollution was associated with T2D^16, 20, 44, 45^, but these previous studies had not corrected for ambient temperature effects. For example, Danish adults who were exposed to higher levels of traffic-related NO_2_ had an increased risk of diabetes^44^. African–American women from Los Angeles who had higher exposure to traffic-related PM2.5 and NO_2_ were also more likely to develop diabetes^20^. Increased levels of PM10 and NO_2_ were also associated with T2D development in adults from Switzerland^16^.

Moreover, our results are in line with previous evidence from animal models^45^. In mice (male C57BL/6) with diet induced obesity, chronic inflammation was suggested to be a mechanism promoting increased insulin resistance after increased PM2.5 exposure^45^. In addition, whole-body glucose homeostasis was reduced with PM2.5 exposure, whereas pro-inflammatory M1 adipose tissue macrophage activity was upregulated and anti-inflammatory M2 adipose tissue macrophage activity was suppressed^45^. Furthermore, it has been suggested that pollutant exposure when superimposed on diabetes and obesity could further aggravate the energy mismatch by triggering immune responses and inducing inflammation^46^. In contrast, lean mice showed little change in insulin sensitivity or lipid profile in response to PM2.5 exposure^45^. This interaction between obesity and PM2.5 in their effects on T2D prevalence was also observed in our analysis.

We found that the effects of PM2.5 on prevalence of T2D and obesity were stronger in females than in males. These findings were also consistent with previous studies^18, 23, 47^. Both biological and non-biological factors are potentially associated with this difference. For example, women have smaller lung size and airway diameter, and it has been suggested that these might increase airway reactivity and exacerbate particulate deposition^48^. However, males have higher metabolic rates than females^49^ and hence the total level of ventilated air into the lungs will be higher in males than in females, suggesting they should perhaps have greater susceptibility to PM2.5 effects. In addition, males spend significantly more time outdoors (8 h) than females (4 h, P < 0.05) further increasing their potential exposure rates^50^. On the other hand, males and females have different socioeconomic status and experiences of life stress^51, 52^. Studies on neighbourhood effects show that women’s health and behaviour are more affected by a number of residential environmental factors^53^.

Although the present article focuses specifically on the relationship between PM2.5 and diabetes, other pollutants not mentioned here have been reported to share a similar relationship with insulin resistance and T2D prevalence^23^. Brook *et al*.^23^ previously reported a relationship between NO_2_ exposure and T2D among patients with respiratory disease in two Canadian cities. It is possible that our analysis has some omitted variable bias, and that other co-pollutants account in part for the relationship between PM2.5 and diabetes. However, spatial correlations between different types of air pollutants in the USA are not particularly strong (30) so this may not be a serious confound.

Previous studies^13, 33, 54, 55^ have noted that PM2.5 and temperature may interact and produce non-additive effects on biological systems. In particular it was noted that cold temperatures exacerbated the impacts of PM2.5 with respect to cardiovascular mortality, for an increase of 10 μg/m^3^ in PM2.5 concentration in the lowest temperature range (−9.7 ∼ 2.6 °C), the relative risk (RR) of cardiovascular mortality increased 1.27% (95% CI 0.38 ∼ 2.17%), which was higher than that of the whole temperature range (0.59%, 95% CI 0.22–1.16%)^13^. In the present study we also observed a weak but significant interaction between the effects of ambient temperature and PM2.5 on T2D prevalence also with low temperatures amplifying the PM2.5 effect. The biological basis of this effect remains uncertain.

Our analysis indicated that PM2.5 had a much greater impact on T2D than on obesity. Hence, other things being equal one would anticipate that the ratio of T2D prevalence to obesity prevalence would increase as PM2.5 increases, and this was indeed the case across the county level data for the USA. The levels of PM2.5 across the continental USA are relatively low when compared with many other regions of the world, and the ratio of T2D to obesity (0.31) is also relatively low. In eastern China and India for example PM2.5 levels are routinely 10x higher than the levels reported in the USA. Plus in both these cases the ratio of T2D to obesity is also very high (China 2.23 and India 2.35). There are however many other differences between the USA and India/China not least of which is the major ethnic difference between the respective populations, which might influence the relative susceptibility of individuals to obesity and diabetes. We sought to explore whether the ratio of T2D to obesity prevalence across countries could be explained by PM2.5 levels. We found that there was indeed a significant effect of PM2.5 on the ratio of T2D to obesity across all 146 countries, supporting the idea that high levels of the T2D to obesity ratio in China and India may in part be driven by high levels of PM2.5.

In conclusion, high levels of PM2.5 probably contribute to increased T2D prevalence in the USA, but have a more minor effect on the prevalence of obesity. Exposure to air pollution may then drive up T2D levels independent of obesity and explain some of the variation in the observed ratio of type 2 diabetes to obesity both within the USA and more globally.
